# Nanocarriers of Miconazole or Fluconazole: Effects on Three-Species *Candida* Biofilms and Cytotoxic Effects In Vitro

**DOI:** 10.3390/jof7070500

**Published:** 2021-06-23

**Authors:** Anne Caroline Morais Caldeirão, Heitor Ceolin Araujo, Laís Salomão Arias, Wilmer Ramírez Carmona, Gustavo Porangaba Miranda, Sandra Helena Penha Oliveira, Juliano Pelim Pessan, Douglas Roberto Monteiro

**Affiliations:** 1 Graduate Program in Dentistry, University of Western São Paulo (UNOESTE), Presidente Prudente 19050-920, SP, Brazil; annemcaldeirao@gmail.com; 2 Department of Preventive and Restorative Dentistry, School of Dentistry, São Paulo State University (Unesp), Araçatuba 16015-050, SP, Brazil; heitor.ceolin@unesp.br (H.C.A.); laisarias@hotmail.com (L.S.A.); wilmer-ramirez.carmona@unesp.br (W.R.C.); juliano.pessan@unesp.br (J.P.P.); 3 School of Dentistry, University of Western São Paulo (UNOESTE), Presidente Prudente 19050-920, SP, Brazil; gporangaba.m@gmail.com; 4 Department of Basic Sciences, School of Dentistry, São Paulo State University (Unesp), Araçatuba 16015-050, SP, Brazil; sandra.hp.oliveira@unesp.br

**Keywords:** antifungals, biofilms, *Candida*, chitosan, cytotoxicity, fluconazole, iron oxide nanoparticles, miconazole, murine fibroblasts, nanocarriers

## Abstract

The contribution of different *Candida* species in oral fungal infections has stimulated the search for more effective therapies. This study assessed the antibiofilm effects of nanocarriers of miconazole (MCZ) or fluconazole (FLZ) on *Candida* biofilms, and their cytotoxic effects on murine fibroblasts. Three-species biofilms (*Candida albicans/Candida glabrata/Candida tropicalis*) were formed on 96-well plates, and they were treated with nanocarriers (iron oxide nanoparticles coated with chitosan—“IONPs-CS”) of MCZ or FLZ at 39/78/156 µg/mL; antifungals alone at 156 µg/mL and artificial saliva were tested as positive and negative controls, respectively. Biofilms were analyzed by colony forming units (CFU), biomass, metabolic activity, and structure/viability. The cytotoxicity (L929 cells) of all treatments was determined via 3-[4,5-dimethylthiazol-2-yl]-2,5 diphenyl tetrazolium bromide (MTT) reduction assay. Data were submitted to one- or two-way ANOVA, followed by Tukey’s or Fisher LSD’s tests (*p* < 0.05). IONPs-CS-MCZ at 78 µg/mL promoted similar antibiofilm and cytotoxic effects compared with MCZ at 156 µg/mL. In turn, IONPs-CS-FLZ at 156 µg/mL was overall the most effective FLZ antibiofilm treatment, surpassing the effects of FLZ alone; this nanocarrier was also less cytotoxic compared with FLZ alone. It can be concluded that both nanocarriers are more effective alternatives to fight *Candida* biofilms compared with their respective positive controls in vitro, being a promising alternative for the treatment of oral fungal infections.

## 1. Introduction

*Candida albicans* is the microorganism isolated in the highest number and at higher frequency from patients with oral mycoses [1,2]. Nonetheless, *Candida glabrata* and *Candida tropicalis* have also been reported to contribute to this condition [1,2]. In addition, infections associated with mixed cultures are of growing concern. In this regard, simultaneous detection of the above-mentioned species has been found in the saliva of dental prosthesis wearers [3], as well as under systemic conditions [4].

Miconazole (MCZ) and fluconazole (FLZ) are antifungal agents commonly used for the topical or systemic treatment of candidiasis, respectively. However, issues associated with these antifungals have been reported, including microbial resistance, hepatotoxicity, local burning sensations, and gastrointestinal disturbances [5,6,7,8]. These limitations clearly indicate the need to improve the pharmacological properties of these antimicrobial agents.

In this sense, the use of nanoparticles as drug carriers has been studied in recent years, aiming to enhance the therapeutic performance of the conjugated compounds and reduce their side effects [9,10]. Recently, our research group assembled nanocarriers of MCZ and FLZ based on chitosan (CS)-coated iron oxide nanoparticles (IONPs), and we found promising antifungal effects on planktonic cells and single- and dual-species biofilms of *C. albicans* and *C. glabrata* [11,12].

Based on the promising effects described above, the present study evaluated the effects of the nanocarriers of MCZ and FLZ on a three-species biofilm model comprising *C. albicans*, *C. glabrata,* and *C. tropicalis*, as well as their cytotoxic effects on murine fibroblasts. The study’s null hypothesis was that the use of the nanocarriers would promote similar antifungal and cytotoxic effects compared with the drugs in their free form.

## 2. Materials and Methods

### 2.1. Assembly and Characterization of the Nanocarriers

To obtain the nanocarriers, MCZ (Sigma-Aldrich, Saint Louis, MO, USA; 500 µg) or FLZ (Sigma-Aldrich; 500 µg) were immobilized on CS (Sigma-Aldrich)-coated IONPs (*n*Chemi Engenharia de Materiais, São Carlos, São Paulo, Brazil) at 700 µg/mL, and they were characterized by several physico-chemical methods, as previously detailed [11,12]. The IONPs-CS-MCZ and IONPs-CS-FLZ nanocarriers showed a predominance of spherical shape and diameters lower than 100 nm, as demonstrated by transmission electron microscopy [11,12].

### 2.2. Effects of the Nanocarriers on Three-Species Candida Biofilms

#### 2.2.1. Microorganisms and Culture Conditions

The following strains from American Type Culture Collection (ATCC) were used in the three-species biofilm model: *C. albicans* ATCC 10231, *C. glabrata* ATCC 90030, and *C. tropicalis* ATCC 750. Microorganisms were separately grown overnight in Sabouraud Dextrose Broth (Difco, Le Pont-de-Claix, France) at 37 °C. Subsequently, the fungal cells were harvested by centrifugation, washed with phosphate-buffered saline (0.1 M, pH 7.0), and adjusted to 1 × 10^7^ cells/mL in artificial saliva (AS) [13], following the protocol used by Arias et al. [12].

#### 2.2.2. Biofilm Formation and Treatment with the Nanocarriers

For three-species biofilm formation, 200 µL of the final inoculum (1 × 10^7^ cells/mL of each yeast) was inserted into 96-well plates (Kasvi, São José dos Pinhais, Paraná, Brazil) and aerobically incubated (48 h) at 37 °C, with partial renewal (50%) of the AS after the first 24 h. After the biofilm formation period (48 h), AS was discarded, and the biofilms were washed once with phosphate-buffered saline. Pre-formed biofilms (48 h) were then treated (24 h) with 200 µL of the nanocarrier suspensions (diluted in AS) containing MCZ or FLZ at 39, 78, and 156 µg/mL. A total of six treatment groups were generated, which were designated as IONPs-CS-MCZ39, IONPs-CS-MCZ78, IONPs-CS-MCZ156, IONPs-CS-FLZ39, IONPs-CS-FLZ78, and IONPs-CS-FLZ156. These concentrations used against biofilms are equivalent to 50-, 100- and 200-fold of the minimum inhibitory concentration value of the nanocarriers for *C. tropicalis* (0.78 µg/mL). Pure MCZ (156 µg/mL) and FLZ (156 µg/mL) were included as controls. The biofilm treated with AS without drugs was designated as the negative control (NC) and processed in the same manner as the treated biofilms.

#### 2.2.3. Biofilm Quantification

The antibiofilm effect of all compounds was assessed by colony-forming units (CFUs) enumeration on CHROMagar *Candida* (Difco) [12]. Semi-quantitative methods of crystal violet (Sigma-Aldrich) staining and 2,3-(2-methoxy-4-nitro-5-sulphophenyl)-5-[(phenylamino) carbonyl]-2H-tetrazolium hydroxide (XTT; Sigma-Aldrich) reduction were also employed to verify the effects on total biomass and metabolic activity, respectively, following protocols detailed in previous studies [14,15]. In turn, the structure and viability of fungal cells were analyzed by confocal laser scanning microscopy (CLSM). For this, biofilms were formed on coverslips (12 mm in diameter; Menzel, Braunschweig, Germany) positioned inside 24-well plates (Kasvi) and submitted to the aforementioned treatments. Next, biofilm samples were stained with SYTO9 and propidium iodide (Filmtracer™ LIVE/DEAD™ Biofilm Viability Kit; Thermo Fisher Scientific, Waltham, MA, USA) following the manufacturer’s instructions, and they were observed under a confocal microscope (Nikon C2/C2si, Tokyo, Japan). Three different areas of CLSM images of each biofilm were analyzed using ImageJ software (version 1.52; Rasband, W.S., ImageJ, U. S. National Institutes of Health, Bethesda, MD, USA) to estimate the percentage of dead cells after treatment with each compound. This estimation was determined by calculating the ratio between the intensity of red fluorescence and total intensity (red + green).

### 2.3. Cytotoxic Effect of the Nanocarriers on Murine Fibroblasts

The cytotoxicity assay was based on the protocol described by Takamiya et al. [16], with minor modifications. Briefly, L929 cells (murine fibroblasts) cultivated (37 °C, 5% CO_2_) in Dulbecco’s modified Eagle’s medium (DMEM; Gibco, Invitrogen Life Technologies, Carlsbad, CA, USA) were seeded in 24-well plates after reaching a monolayer with 90–100% confluence. After, fibroblastic cells adhered to the bottom of the wells were exposed for 24 and 48 h to different concentrations of MCZ (0.24–250 μg/mL) and FLZ (0.48–1000 μg/mL), alone or forming the nanocarriers (IONPs-CS-MCZ and IONPs-CS-FLZ). Cell viability was measured by the colorimetric 3-[4,5-dimethylthiazol-2-yl]-2,5 diphenyl tetrazolium bromide (MTT) reduction assay [17], and the results were represented as percentage of cell viability in relation to NC (cells treated with pure DMEM).

### 2.4. Statistical Analysis

All microbiological and cell culture assays were conducted on three separate occasions, each in triplicate. Biofilm and cytotoxicity data passed the normality test (Shapiro–Wilk) and were examined by one- and two-way ANOVA, respectively. For multiple comparisons among groups, Fisher LSD’s test was applied for biofilm results, while Tukey’s test was used for cytotoxicity data. Statistical analyses were performed using the SigmaPlot software (version 12.0; Systat Software Inc., San Jose, CA, USA) at a significance level of 5%.

## 3. Results

### 3.1. Effects of the Nanocarriers on Three-Species Candida Biofilms

**Figure 1:**
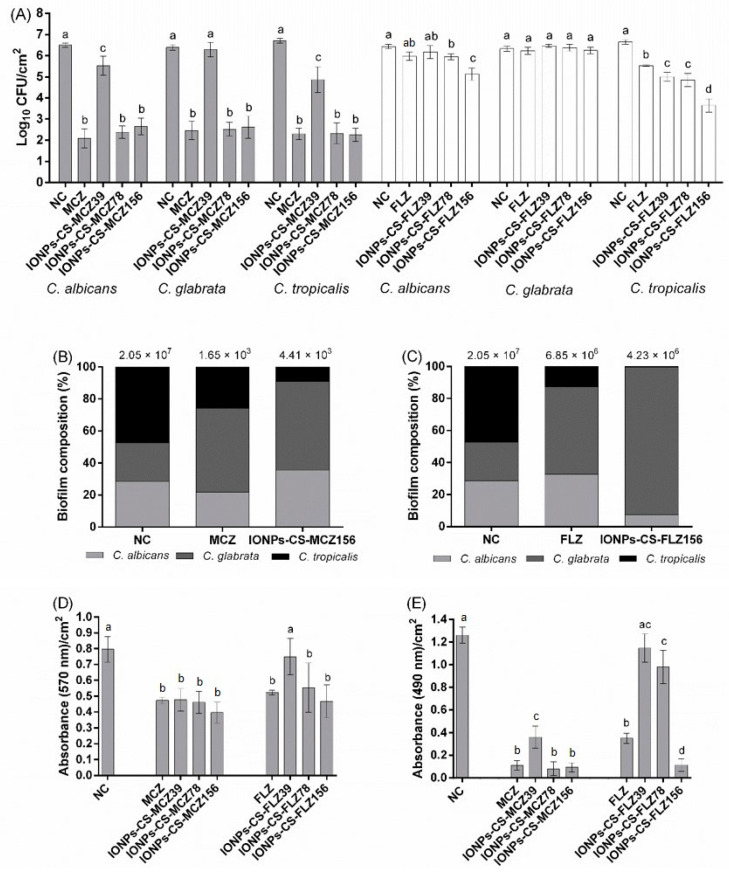
Colony-forming units quantification (**A**), compositional analysis (**B**,**C**), total biomass (**D**), and metabolic activity (**E**) of 48 h mixed biofilms of *Candida albicans*, *Candida glabrata*, and *Candida tropicalis* after treatment with different compounds. Biofilms were treated during 24 h with miconazole (MCZ) at 156 µg/mL, chitosan (CS)-coated iron oxide nanoparticles (IONPs) carrying MCZ at 39 (IONPs-CS-MCZ39), 78 (IONPs-CS-MCZ78), and 156 µg/mL (IONPs-CS-MCZ156), fluconazole (FLZ) at 156 µg/mL, and FLZ-containing nanocarrier at 39 (IONPs-CS-FLZ39), 78 (IONPs-CS-FLZ78), and 156 µg/mL (IONPs-CS-FLZ156). Biofilms grown for 72 h in artificial saliva devoid of drugs depict the negative control (NC). Distinct lower-case letters depict significant differences among the groups within each drug (one-way ANOVA and Fisher LSD’s test; *p* < 0.05). Statistical analyses were conducted separately for each antifungal agent, its nanocarriers, and NC.

MCZ, IONPs-CS-MCZ78, and IONPs-CS-MCZ156 did not differ from one another and achieved the greatest reductions (3.77- to 4.46-log_10_; *p* < 0.001) in CFU numbers of *C. albicans*, *C. glabrata*, and *C. tropicalis* compared to NC (Figure 1A). Although IONPs-CS-MCZ39 led to significant decreases in CFUs of *C. albicans* and *C. tropicalis* compared to NC, this nanocarrier was not able to overcome the reductions promoted by MCZ alone (Figure 1A).

IONPs-CS-FLZ156 was the most effective compound in reducing CFUs of *C. albicans* (1.31-log_10_; *p* < 0.001) and *C. tropicalis* (3.03-log_10_; *p* < 0.001) compared to NC, differing significantly from all other groups (Figure 1A). In addition, IONPs-CS-FLZ78 significantly decreased CFUs of *C. albicans* (reduction of 0.48-log_10_; *p* = 0.026) and *C. tropicalis* (reduction of 1.82-log_10_; *p* < 0.001) compared to NC, surpassing the effects achieved by FLZ alone for the latter. For *C. glabrata*, however, no compound was able to affect the number of CFUs (Figure 1A).

The compositional analysis revealed that *C. tropicalis* was the most prevalent species in the NC group, representing 47.23% of the total biofilm cells, followed by *C. albicans* (28.66%) and *C. glabrata* (24.10%) (Figure 1B,C). However, after treatments with IONPs-CS-MCZ156, FLZ, and IONPs-CS-FLZ156, *C. tropicalis* became the least prevalent species in the biofilm, comprising 8.9, 12.7, and 0.3% of the total cells, respectively. An inverse behavior was observed for *C. glabrata*, which became the most prevalent species after all treatments, mainly for IONPs-CS-MCZ156 and IONPs-CS-FLZ156, representing 55.4 and 92.2% of the biofilm cells, respectively (Figure 1B,C). For *C. albicans*, its prevalence increased after treatment with IONPs-CS-MCZ156 (35.7% of the total cells) and FLZ (32.7% of the total cells), and decreased after exposure to MCZ (21.9% of the total cells) and IONPs-CS-FLZ156 (7.5% of the total cells), all compared to NC (Figure 1C).

As for total biofilm biomass, MCZ and its nanocarriers did not differ from each other and significantly reduced the total biomass compared to NC (41.3–51.3%; *p* < 0.001) (Figure 1D). FLZ, IONPs-CS-FLZ78, and IONPs-CS-FLZ156 also significantly decreased the total biomass compared to NC, with reductions ranging from 31.3 to 41.3% (*p* ≤ 0.007; Figure 1D). For metabolic activity, all compounds led to significant reductions compared to NC (*p* ≤ 0.001), except IONPs-CS-FLZ39 (Figure 1E). It should be noted that IONPs-CS-FLZ156 led to a 91.3% reduction in metabolic activity compared to NC, significantly exceeding (*p* = 0.004) the effect generated by FLZ in its free form (reduction of 72.2%).

**Figure 2:**
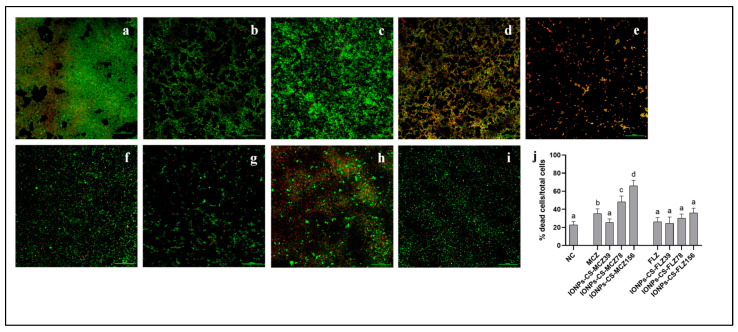
Confocal laser scanning microscopy images of 48 h mixed biofilms of *Candida albicans*, *Candida glabrata*, and *Candida tropicalis* treated (24 h) with miconazole (MCZ) at 156 µg/mL (**b**), chitosan (CS)-coated iron oxide nanoparticles (IONPs) carrying MCZ at 39 (**c**), 78 (**d**), and 156 µg/mL (**e**), fluconazole (FLZ) at 156 µg/mL (**f**), and FLZ-containing nanocarrier at 39 (**g**), 78 (**h**), and 156 µg/mL (**i**). Biofilm grown for 72 h in artificial saliva devoid of drugs depicts the negative control (NC;**a**). Dead and living cells are represented by red and green fluorescence, respectively. Magnification: 20×. Bars: 100 µm. The percentages of dead cells after treatment with each compound are shown in image “**j**”. Distinct lowercase letters depict significant differences among the groups within each drug (one-way ANOVA and Fisher LSD’s test; *p* < 0.05). Statistical analysis was conducted separately for each antifungal agent, its nanocarriers, and negative control.

Regarding biofilm structure, all groups were comprised of yeast cell clusters covering the surface of the coverslips (Figure 2). The NC group showed a more compact and dense architecture compared to the other groups. In turn, IONPs-CS-MCZ156 promoted the greatest disruption in biofilm structure, being composed of sparse yeasts forming a less dense architecture compared to NC (Figure 2e). Although MCZ led to a higher percentage of dead cells compared to NC (*p* = 0.014), its reducing effect on cell viability was significantly exceeded by IONPs-CS-MCZ78 (*p* = 0.011) and IONPs-CS-MCZ156 (*p* < 0.001; Figure 2j). The highest percentage of dead cells was observed after treatment with IONPs-CS-MCZ156 (66.1%), significantly differing from the other groups (*p* ≤ 0.002; Figure 2j). On the other hand, FLZ and its nanocarriers were not able to reduce cell viability compared to NC (Figure 2j).

### 3.2. Cytotoxic Effect of the Nanocarriers on Murine Fibroblasts

**Figure 3:**
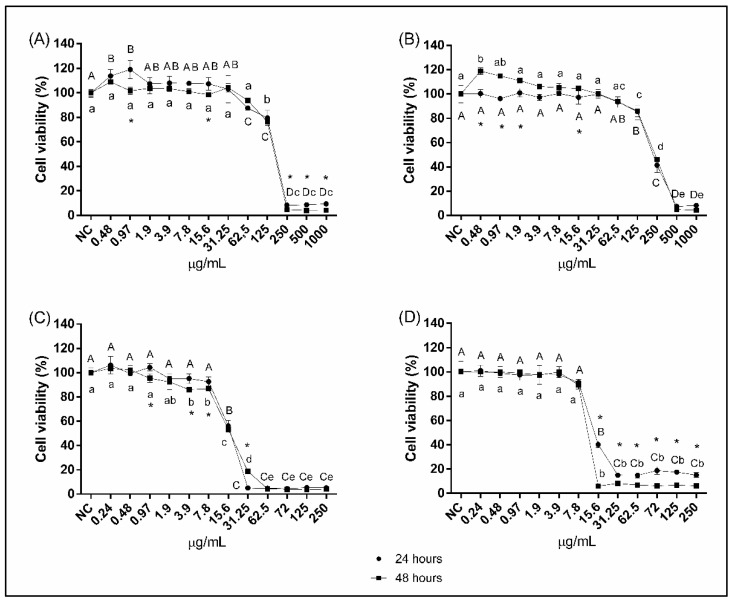
Percentages of viable L929 cells (murine fibroblasts) after exposure to fluconazole at 0.48–1000 µg/mL (**A**), fluconazole-containing nanocarrier at 0.48–1000 µg/mL (**B**), miconazole at 0.24–250 µg/mL (**C**), and miconazole-containing nanocarrier at 0.24–250 µg/mL (**D**). Fibroblasts cultivated in drug-free culture medium depict the negative control (NC). Different upper- and lower-case letters indicate significant differences among the concentrations of the compounds, respectively, for 24 and 48 h of exposure. * Represents significant difference between 24 and 48 h of exposure, within each concentration assessed (two-way ANOVA and Tukey’s test; *p* < 0.05).

After exposure to FLZ for 24 and 48 h, cell viability was not altered at concentrations equal to or lower than 31.25 and 62.5 μg/mL, respectively (Figure 3A). However, at concentrations equal to or greater than 250 μg/mL, dramatic reductions in viability (>90%) were observed, regardless of the exposure period (Figure 3A). For the IONPs-CS-FLZ nanocarrier, concentrations similar to pure FLZ did not alter cell viability (Figure 3B). In turn, concentrations equal to or greater than 500 μg/mL dramatically reduced the cell viability compared to the control group, for both 24 and 48 h of exposure (Figure 3B). Regarding MCZ, doses equal to or lower than 7.8 μg/mL did not alter the cell viability after 24 h of contact (Figure 3C). However, after 48 h of exposure, concentrations equal to or greater than 3.9 μg/mL significantly reduced the cell viability (Figure 3C). Reductions greater than 81.3% were observed in the concentration range from 31.3 to 250 μg/mL, for both periods of exposure (Figure 3C). For the IONPs-CS-MCZ nanocarrier, concentrations equal to or lower than 7.8 μg/mL were not cytotoxic (Figure 3D). Comparing the two exposure periods in the concentration range from 15.6 to 250 μg/mL, the cytotoxicity of the nanocarrier was significantly higher after 48 h of exposure (Figure 3D).

## 4. Discussion

In the present study, the antifungal and cytotoxic effects of MCZ- and FLZ-nanocarriers were evaluated on mixed biofilms of three *Candida* species and on murine fibroblasts, respectively. The null hypotheses were partially rejected, as IONPs-CS-FLZ156 promoted significantly higher antibiofilm effects and lower cytotoxic effects compared with pure FLZ. In turn, IONPs-CS carrying MCZ at 78 or 156 µg/mL did not affect the antibiofilm and cytotoxic effects compared with MCZ alone.

For cultivable cell quantification assays, IONPs-CS-MCZ78 promoted similar antibiofilm effects as pure MCZ and IONPs-CS-MCZ156 (Figure 1A). This attests the advantage of nanocarriers, which can reduce the effective concentration of a drug, thereby minimizing possible side effects associated with the use of certain drugs. This favorable antibiofilm action achieved by IONPs-CS-MCZ78 may result from an additive effect promoted by the compounds that form the nanocarrier.

Following this reasoning, IONPs can lead to microbial cell death through their binding to cell membranes, which promotes depolarization and loss of membrane integrity, in addition to extravasation of intracellular constituents [18]. Moreover, these nanoparticles stimulate the formation of reactive oxygen species (ROS) with lipid peroxidase, causing damage to deoxyribonucleic acid and cellular proteins [18,19], as well as causing the loss of cell homeostasis due to the presence of ions [18]. Despite their good biological and physical-chemical properties, IONPs must be functionalized to prevent their agglomeration and to improve their surface activity and biocompatibility [18]. In this context, the coating of IONPs with CS favors the stability of nanoparticles and provides a positive charge to the compound IONPs-CS, which can favor the interaction with negatively charged cell membranes [18,19]. CS also has mucoadhesive properties, which facilitate its retention on target cells [18]. In turn, the mechanism of antifungal action of MCZ comprises the inhibition of the enzyme lanosterol 14-alpha-demethylase, which participates in the ergosterol synthesis of the fungal membrane [20].

Regarding the nanocarriers of FLZ, IONPs-CS-FLZ156 also promoted significant reductions in CFUs of *C. albicans* and *C. tropicalis*, overcoming the effect of FLZ alone (Figure 1A). These results are also indicative of the cooperative action among the nanocarrier compounds, as discussed above. Concerning the mechanism of action of FLZ, this antifungal agent inhibits the synthesis of ergosterol and enzymes of the cytochrome P-450 complex (including CYP34A), affecting fungal membrane fluidity, and cell growth and proliferation [21]. It should be noted that IONPs-CS-FLZ was not able to overcome the problem of intrinsic resistance of *C. glabrata* to FLZ, as detailed in previous studies [22,23], considering that FLZ (free or conjugated to the compound IONPs-CS) did not reduce CFUs of *C. glabrata* (Figure 1A). In this sense, a previous study showed that FLZ and IONPs-CS-FLZ were effective in reducing cultivable cells of single biofilms of *C. albicans* and *C. glabrata* [11], though at a FLZ concentration (1250 µg/mL) 8–32 times higher than those used in the present study. This comparison demonstrates that IONPs-CS-FLZ works effectively at lower concentrations of the antifungal in cases of co-infection by *C. albicans*, *C. glabrata*, and *C. tropicalis*, under a condition that better resembles the context of the oral microbiome in cases of candidiasis. In contrast to the results found here, IONPs-CS-MCZ78 was shown to overcome the reducing effect of the free drug on *C. albicans* and *C. glabrata* in single or mixed biofilms of the two species [12]. In view of these differences, a greater tolerance of *Candida* species to MCZ can be inferred when in multispecies interaction.

In relation to compositional analysis, *C. tropicalis* was the most predominant species in the untreated biofilm, whereas after treatment with antifungals and their nanocarriers, this scenario was changed, with *C. glabrata* being the most prevalent species (Figure 1B,C). For FLZ and IONPs-CS-FLZ156 (Figure 1C), these results are in line with those of CFU and prove that the lack of a reducing effect on *C. glabrata* resulted in its higher prevalence in the biofilm after treatment. However, for MCZ and IONPs-CS-MCZ156 (Figure 1B), a direct relationship between CFU results and compositional analysis was not observed. In this case, it is likely that the highest prevalence of *C. glabrata* is associated with the interactions established between the different species. Under conditions of co-infection, the different biofilm species can establish synergistic or antagonistic relationships [24]. Therefore, it is possible to infer that *C. glabrata* cells were protected by *C. albicans* and *C. tropicalis*, which contributed to their higher prevalence in biofilms treated with MCZ and IONPs-CS-MCZ156.

All compounds evaluated in this study significantly reduced the total biomass and metabolic activity of the biofilms compared to the NC, except IONPs-CS-MCZ39 and IONPs-CS-FLZ39 (Figure 1D,E). These results are related to those of CFUs, considering that cell death may have led to a reduction in metabolic activity and, consequently, in the production of extracellular matrix, directly affecting the total biomass.

CLSM analysis revealed that IONPs-CS-MCZ156 promoted the largest disruption in the biofilm structure, and significantly increased the number of dead cells compared to the other groups, and that the effectiveness of IONPs-CS-MCZ is directly proportional to the concentration of MCZ (Figure 2). These findings also show that the nanocarrier of MCZ can overcome the antibiofilm effect of MCZ in its free form. Although the CLSM results contrast with those presented in Figure 1, these apparent inconsistencies may be associated with the types of surfaces on which the biofilms were formed in the different tests.

Overall, the MTT assay revealed similar cytotoxicity rates for each antifungal and their respective nanocarriers, regardless of the exposure time (Figure 3). Although a previous study found that IONPs and CS alone led to marked reductions in the viability of L929 cells for concentrations from 87.5 and 175 µg/mL, respectively [25], these compounds did not potentiate the cytotoxic effects of the IONPs-CS-FLZ and IONPs-CS-MCZ nanocarriers. In fact, the results of the present study indicate that the nanocarriers’ cytotoxicity is primarily dependent on the presence of the antifungals (FLZ and MCZ), corroborating a previous study in which the cytotoxicity of a cetylpyridinium chloride nanocarrier was also dependent on the presence of the drug, with no influence of the carrier compound (IONPs-CS) [26].

The cytotoxicity mechanisms of FLZ and MCZ have also been reported to depend on the cell line tested. The treatment of L929 cells with FLZ (250–1000 µg/mL) was not shown to affect the cell apoptosis rate compared to the untreated control group, despite reducing the concentration of total proteins in the culture medium as a consequence of reductions in the cell proliferation rate [27]. On the other hand, FLZ is able to induce the death of monkey kidney cells by necrosis, as it leads to membrane rupture and the leakage of intracellular content [28]. FLZ may also induce an increase in ROS and generate DNA damage [28]. In turn, MCZ (12.5 µg/mL) stimulates the production of ROS in human keratinocytes (HaCaT), which may induce oxidative stress and cause cell death [29]. In addition, MCZ inhibits the growth of bladder cancer cells by activating extrinsic and intrinsic apoptotic pathways [30].

It should also be emphasized that L929 cells exposed to FLZ at concentrations of 125 and 250 µg/mL showed less viability (between 4.8 and 79.6%) compared to the IONPs-CS compound carrying FLZ at the same concentrations (between 41.3 and 85.7%) (Figure 3A,B). These findings, in conjunction with the biofilm results, indicate a double advantage of IONPs-CS carrying FLZ at 156 µg/mL: lower cytotoxic potential and better antibiofilm effect in reducing the metabolic activity and the count of CFUs of *C. albicans* and *C. tropicalis* compared to FLZ (Figure 1). In turn, for MCZ and its nanocarriers, IONP-CS-MCZ78 showed an antibiofilm effect similar to that achieved by MCZ at a two-fold higher concentration (i.e., 156 µg/mL). However, this nanocarrier did not reduce the cytotoxic potential compared with free MCZ.

## 5. Conclusions

The administration of MCZ or FLZ as nanocarriers was able to enhance the effects against *Candida* species in co-culture biofilms compared with the antifungals in their free form, with effects depending on the type and concentration of each antifungal, *Candida* species, and type of variable analyzed. Marked reductions in the cytotoxic potential of IONPs-CS-FLZ156 were also observed compared with FLZ at the same concentration. Given that the antibiofilm effects were achieved either by potentiating the inhibitory action compared with its free counterpart (free FLZ), or promoting similar antibiofilm effects using half of the concentration of the free drug (MCZ), the results reported here open avenues for the development of dual nanocarrier systems as alternative nanotherapies against oral fungal infections.

## Data Availability

The data presented in this study are available on request from the corresponding author.
